# Special Issue: Autoimmune Diseases: A Swing Dance of Immune Cells

**DOI:** 10.3390/ijms26199365

**Published:** 2025-09-25

**Authors:** Balik Dzhambazov

**Affiliations:** Department of Developmental Biology, Faculty of Biology, Paisii Hilendarski University of Plovdiv, 24 Tsar Assen Str., 4000 Plovdiv, Bulgaria; balik@uni-plovdiv.bg; Tel.: +359-32-261-535

Autoimmune disorders occur when the body’s immune defenses misidentify its own tissues as threats, disrupting the normal mechanisms that preserve immune self-recognition. This loss of immune harmony is often compared to a dance gone awry, where partners that usually move in synchrony (immune cells) become misaligned. This immune coordination reflects a dynamic interaction between inherited genetic factors, environmental influences, and a diverse range of immune cells [1]. In conditions such as rheumatoid arthritis (RA) or systemic lupus erythematosus (SLE), for example, many arms of the immune system—innate cells (macrophages, neutrophils, and dendritic cells) and adaptive cells (T and B lymphocytes)—are activated and contribute to widespread inflammation and tissue damage [2]. The result is a self-destructive immune response that can affect specific organs or multiple systems. This Special Issue particularly focuses on restoring balance in this “swing dance” of immune cells, covering various topics from understanding the basic mechanisms of immune regulation to developing novel diagnostic tools and therapies.

The first edition of this Special Issue, “Autoimmune Diseases: A Swing Dance of Immune Cells” (https://www.mdpi.com/journal/ijms/special_issues/RH7MHGH5JD, accessed on 23 September 2025), highlighted that autoimmune diseases cannot be understood solely as T- or B-cell misfires. Instead, they should be seen as disorders of the entire tissue ecosystem, where barriers, stromal cells, and immune effectors interact through tightly linked feedback loops that influence both disease progression and treatment possibilities. The main scientific insights were as follows:

- Barrier tissues prime and mirror autoimmune pathology, presenting diagnostic and therapeutic opportunities at sites presenting early signs of disease.
- Stromal cells act as immune selectors, linking tissue chemistry to adaptive repertoire shaping.
- Nodal molecules with dual roles, such as galectin-3 and αvβ3, represent high-value therapeutic targets.
- Reinstating regulatory cytokine tone (e.g., IL-35) may counter fibrosis and inflammation.
- The pathogenesis of rheumatoid arthritis involves multicellular units that require integrated therapeutic strategies.

In the current (second) edition of this Special Issue (https://www.mdpi.com/journal/ijms/special_issues/X9RN2VMM1Z, accessed on 23 September 2025), articles, authored by researchers across various fields, illuminate how missteps in the immune dance lead to autoimmunity and how we could correct them.

Fiyouzi et al. (Contribution 1) explored a novel strategy to enhance immune regulation by harnessing regulatory T cells (Tregs), identifying specific peptide epitopes derived from the self-protein α-tubulin that can induce functional Tregs. The authors performed in vitro experiments demonstrating that a pool of α-tubulin peptides increased IL-10-producing FoxP3^+^ Tregs and type 1 regulatory T (Tr1) cells while suppressing conventional T cell responses. Moreover, they stimulated human naive CD4^+^ T cells using dendritic cells in the presence of these peptides, promoting their differentiation into FoxP3^+^ Tregs, which can suppress the proliferation of effector T cells. These findings suggest that widely expressed proteins such as α-tubulin harbor epitopes that can “tune” the immune dance toward tolerance. By increasing the population of Tregs, such peptides, or even peptides identified through computational mapping tools [3], could help control excessive or misdirected immune responses, offering a promising avenue for treating autoimmune conditions.

Lacka et al. (Contribution 2) provided a genetic study on autoimmune thyroiditis, specifically Hashimoto’s thyroiditis, one of the most prevalent organ-specific autoimmune diseases. They examined a single-nucleotide polymorphism in the thyroid peroxidase gene (TPO rs1126797) to assess its contribution to disease susceptibility and phenotype in a Caucasian Polish cohort. TPO is a key thyroid autoantigen (a target of anti-thyroid antibodies in Hashimoto’s disease) and a possible candidate gene for predisposition to the disease. The study indicated no significant association between TPO rs1126797 polymorphism and the risk of autoimmune thyroiditis in this population. The allele and genotype frequencies of rs1126797 were similar in patients and healthy controls, a finding that aligns with earlier studies on other ethnic groups. Overall, this TPO variant does not exhibit a strong effect, reinforcing the suggestion that autoimmune thyroiditis is multifactorial, with many genetic and environmental factors each exerting some influence [4]. Larger, ethnically diverse studies will be needed to confirm whether any genotype–phenotype correlations (such as thyroid size or antibody levels) exist for rs1126797.

Taking autoimmune attacks on the central nervous system as their research focus, Takano et al. (Contribution 3) investigated the role of hepatocyte growth factor (HGF) in multiple sclerosis (MS) pathology. HGF is a pleiotropic cytokine, known as a key factor preventing neuronal death, that induces neuroregeneration and immunoregulatory functions [5]; in MS patients on immunomodulatory therapy, its levels increase. Using experimental autoimmune encephalomyelitis (EAE), a murine model of MS, the researchers showed that neuron-derived HGF can ameliorate neuroinflammation. Transgenic mice overexpressing HGF exhibited a delayed onset and reduced severity of EAE compared to wild-type mice. Neuropathological analysis revealed that HGF overexpressors experienced less demyelination and axonal degeneration in the spinal cord, with electron microscopy confirming that myelin integrity was better preserved. Mechanistically, HGF’s beneficial effects were associated with the modulation of key receptors—the proinflammatory receptor c-Met was less upregulated in HGF-Tg mice during EAE, whereas levels of KAI-1 (CD82), a molecule linked to cell adhesion and axonal protection, were elevated. Together, the findings underscore HGF’s potential as a neuroprotective and immunomodulatory agent in MS—essentially helping immune cells and glia “dance” more harmoniously. By preserving CNS structure and function in the face of autoimmunity, HGF and its signaling pathways (c-Met/KAI-1) show promise as therapeutic targets that can slow the progression of MS.

Nötzel et al. (Contribution 4) introduced an innovative diagnostic approach, applying Raman spectroscopy to profile immune cells in a label-free, non-invasive manner. Given immune cell states (e.g., the activation of T cells) often need to be monitored in autoimmune diseases, this technique offers a potential new “biomarker” strategy by capturing the molecular vibrations (chemical fingerprint) of cells. The authors used Raman spectroscopy to analyze optically trapped living immune cells, aiming to differentiate different T cell subsets and activation states. The spectroscopic signatures could distinguish between naïve versus CD3/CD28-stimulated human T cells for both CD4^+^ and CD8^+^ lineages, reflecting underlying biochemical changes upon T cell activation. Using multivariate analysis (principal component and linear discriminant analysis), the authors could classify cell types with a good level of accuracy based on their spectra—monocytes were identified with ~83% accuracy, while CD4^+^ and CD8^+^ T cells showed distinct profiles from their activated counterparts. These results expand our knowledge of immune cell “fingerprints” and pave the way for future clinical applications. In principle, Raman spectroscopy could be utilized to monitor the activation of immune cells in patients with autoimmune diseases or rapidly diagnose abnormalities of immune cells, all without the need for fluorescent markers or extensive sample processing.

Zhang et al.’s study (Contribution 5) examined whether the “sex” of donor plasma affects the therapeutic efficacy of intravenous immunoglobulin (IVIG) in autoimmune disease. IVIG, a pooled IgG product from thousands of donors, is a first-line therapy for many autoimmune conditions, including immune thrombocytopenia (ITP), where it can block the destruction of autoantibody-mediated platelets. The authors investigated whether the efficacy of IVIG therapy differed based on the donor’s sex, as there are known differences in the composition of plasma proteins between males and females and the prevalence of autoimmune disease demonstrates sex-related differences. They prepared three formulations of IVIG, from female-only plasma (F-IVIG), male-only plasma (M-IVIG), or a standard mixed pool (Mix-IVIG), and tested them in a mouse model of ITP. Notably, M-IVIG was the most effective in alleviating ITP-related measures. Mice that received M-IVIG exhibited the most improved platelet counts and spleen indices (a readout of splenic inflammation) compared to those given female-derived or mixed IVIG. All IVIG preparations provided protection in mice with ITP by mitigating inflammation, but the male donor IVIG outperformed the female donor IVIG in several key immunological parameters. This novel finding suggests that biological differences between male and female plasma (such as levels of certain IgG subclasses, cytokines, or accessory proteins) can influence the efficacy of IVIG therapy. This raises practical considerations for the production of IVIG and personalized medicine—in the future, tailoring immunoglobulin products (or donor selection) could optimize treatments for autoimmune patients.

Anton et al. (Contribution 6) provided a comprehensive review on the interplay between the lungs and rheumatoid arthritis. RA is well known for causing joint inflammation, but it also manifests in other organs, with interstitial lung disease (ILD) constituting one of the most serious extra-articular complications, contributing to increased morbidity and mortality in patients with RA. The authors outline how the lungs can serve as both a target organ damaged by systemic autoimmune processes and a possible site where autoimmune processes begin. Environmental factors such as smoking or silica exposure can cause local lung irritation and the citrullination of proteins in the respiratory mucosa. In genetically susceptible individuals, these altered proteins may break immune tolerance, leading to the production of anti-citrullinated protein antibodies (ACPAs)—a hallmark of RA. The study also outlines emerging evidence that, in some patients with RA, the autoimmune response may begin in the lungs: ACPAs and other autoantibodies may be initially generated in pulmonary lymphoid tissue and then “migrate” or disseminate to the joints, where they induce synovial inflammation. Additionally, in RA, systemic inflammation is driven by various immune cells (such as macrophages, Th1/Th17 cells, and B cells) that can move to lung tissue, causing chronic inflammation and fibrosis in susceptible individuals. This review thus bridges pulmonary medicine and rheumatology, emphasizing that autoimmune diseases often involve multi-organ crosstalk.

Loriamini et al. (Contribution 7) delivered an extensive review on autoimmune hemolytic anemias (AIHAs)—disorders where the immune system attacks red blood cells (RBCs), leading to their premature destruction. This contribution serves as a crucial reference for both researchers and clinicians, as it consolidates current knowledge on the classifications, pathophysiological mechanisms, diagnostic workup, and management of AIHAs. The authors categorize AIHAs into several types according to presence of autoantibodies and lab findings (particularly results from the direct antiglobulin test, DAT). In terms of treatment, the review highlights that managing AIHA frequently requires immunosuppressive therapy to reduce the production or function of autoantibodies. First-line therapy for warm AIHA is corticosteroids, with high response rates. Refractory cases may require steroid-sparing agents or B-cell depletion (e.g., rituximab targeting CD20). Cold AIHA management involves keeping patients warm and using agents such as rituximab or complement inhibitors (since the complement system plays an important role in cold agglutinin hemolysis). Overall, the review acts as a comprehensive guide through the “dance floor” of autoantibody–RBC interactions, from classification to cutting-edge treatments.

In summary, the articles in this Special Issue collectively illustrate the multifaceted nature of autoimmunity and ongoing efforts to restore harmony in the immune system’s “swing dance.” Several common themes emerge, as listed below:

1. Immune regulation is key: Approaches that enhance the body’s own regulatory mechanisms (such as Treg-inducing peptides or tissue-protective factors such as HGF) could dampen autoimmune pathology. By increasing regulatory T cells or protective cytokine signaling, these strategies aim to correct the missteps in immune responses before irreversible damage occurs.
2. Patient-specific factors matter: Genetic variations or donor-related differences, as shown by the TPO polymorphism study and the sex-specific IVIG study, can influence the expression of disease and efficacy of treatment. This highlights the importance of personalized medicine—understanding an individual’s genetic makeup or the composition of therapeutics could inform tailored interventions to achieve better outcomes.
3. Innovative technologies and comprehensive analyses can enhance the diagnosis and classification of autoimmune diseases. The Raman spectral profiling of immune cells exemplifies how novel diagnostics can be used to detect immune activation states in a label-free manner, whereas extensive reviews (of RA-ILD and AIHA) synthesize vast data that can guide clinicians in recognizing and managing complex disease manifestations.
4. The “dance” of immune cells in autoimmunity often spans multiple organs and systems. The interplay between the lungs and joints in RA and between the immune system and the hematologic system in AIHA reminds us that autoimmune diseases are not isolated to one cell type or tissue—they are systemic disorders of immune miscommunication.

Overall, we can surmise that effective solutions require an integrated perspective. By simultaneously targeting immune overactivity (e.g., via induced Tregs or B-cell modulation), considering personal/genetic factors, and employing precise diagnostics, we move closer to re-establishing immune tolerance without compromising necessary immunity. Ongoing and future research inspired by these findings will continue to waltz us forward, turning cacophony into choreography and discord into a well-tuned swing dance of immune cells where the rhythm of self-tolerance is finally restored.
